# The Future of Orthopaedic Education and Training: Embracing Innovation for a Changing Landscape

**DOI:** 10.7759/cureus.67146

**Published:** 2024-08-18

**Authors:** Vinod Nair, Amogh Todkar, Harsh Kumar

**Affiliations:** 1 Orthopaedics, Dr. D.Y. Patil Medical College Hospital and Research Centre, Dr. D.Y. Vidyapeeth (Deemed to be University), Pune, IND

**Keywords:** simulation, inovative technology, skills laboratory, virtual reality, orthopaedics

## Abstract

Technological, surgical, and patient demographic developments are driving a perpetual state of change in the field of orthopaedics. Orthopaedic education and training must evolve to better educate upcoming generations of surgeons for this changing environment if the profession is to continue to flourish. The promise of cutting-edge teaching techniques, such as virtual reality (VR), online learning environments, and simulation technology, is examined in this editorial, along with the necessity of developing curricula that take into account patients' evolving requirements and new technological advancements.

## Editorial

In order to help patients with musculoskeletal diseases live better lives, orthopaedic surgeons are important. The conventional approach to orthopaedics education and training, however, finds it difficult to keep up with the field's quick advances. Cadaver labs, supervised clinical rotations, and didactic lectures are major components of residency programs. Although these approaches are still important, adding cutting-edge technologies and adaptable learning options will greatly improve the quality of education.

Beyond the foundational elements of lectures and cadaver labs, the future of orthopaedic education lies in embracing innovative technologies.

Simulation technology

Simulation-based surgical skills training is widely recognized as a valuable method for improving trainees' performance and as an essential part of a comprehensive curriculum in surgical education [1]. Trainers can practice surgical operations and refine essential decision-making abilities in a safe and controlled environment with simulators that replicate real-world circumstances with great fidelity. This keeps patient safety in check while enabling them to learn from their mistakes [2].

Virtual reality

Understanding the anatomical orientation of joints such as the shoulder, knee, and ankle can be really challenging for residents. Here, virtual reality (VR) offers genuinely immersive experiences that surpass mere simulation. In a risk-free setting, trainees can enter virtual operating rooms, practice intricate surgical procedures on three-dimensional models, and run into unanticipated difficulties. Various specialties in othropaedics, such as arthroplasty, arthroscopy, foot, and ankle, need some exposure during the duration of orthopaedic residency, which many fail to experience due to various reasons. This challenge can be bridged by VR by providing an orientation into those specialties, which would help the residents choose the correct specialty for them. Arthroscopic simulators, such as the Knee Arthroscopy Surgical Trainer (KAST), developed by the American Academy of Orthopaedic Surgeons (AAOS), and the Simbionix Arthromentor, are capable of providing haptic feedback on simulated cartilage and tendon and mimic surgical tools [3]. Virtual reality enables practice and proficiency with procedures prior to going into the operating room. Consequently, VR simulations offer unique advantages in comparison to more traditional simulators and other methods of education [4].

Online learning platforms

Digitization in orthopaedics and traumatology is an enormously fast-evolving field with numerous players and stakeholders. It will be of utmost importance that the different groups of technologists, users, patients, and actors in the healthcare systems learn to communicate in a language with a common basis [5]. With the flexibility and accessibility these platforms provide, learners may conveniently revisit course materials and progress at their own speed. An extensive range of subjects, from fundamental anatomy to sophisticated surgical methods, can be covered by online courses, which promote independent learning and knowledge retention.

A common platform can be created online where all the stalwarts of orthopaedics teachers can teach their years of experience, pass on surgical knowledge, and also guide the resident doctors on how to choose the right path in the dynamic field of orthopaedics. This would be a game-changing experience for the residents. These classes or videos will be available across the internet to the most remote areas and will be accessible to everybody at any time and anywhere.

Skills laboratories play a vital role in bridging the knowledge gap between academic theory and the development of practical abilities. These specialized facilities offer a realistic setting furnished with surgical tools, models, and visual aids. Here, students are able to practice essential skills, from suturing and knot-tying to bone reduction and implant handling. Skills laboratories allow for hands-on practice under the guidance of experienced instructors. Skills laboratories can be used to deconstruct complex surgeries into smaller, manageable steps that trainees or residents can practice and perfect before performing them on real patients, helping the resident doctors and trainees master procedural steps. Skills laboratories often incorporate team-based exercises, fostering collaboration and communication skills essential for successful surgical teams. 

While innovative educational methods such as simulation, virtual reality, and online learning offer significant advantages in orthopaedic education and training, they also present certain challenges and potential disadvantages, such as high costs of infrastructure and content development. Implementing simulation labs and VR technology requires substantial financial investment in equipment, software, and maintenance. Creating high-quality simulation scenarios and online courses demands significant time, expertise, and resources. Technical difficulties with simulation equipment or online platforms can disrupt learning and training. Access to technology and internet connectivity may vary among trainees, creating disparities in learning opportunities. Simulation and virtual environments may not fully replicate the tactile sensations of real-life surgical procedures.

Excessive reliance on simulation could potentially reduce opportunities for hands-on surgical experience. Online learning and independent simulation practice can lead to reduced interaction with peers and mentors and can lead to isolation and reduced interaction. As trainees may not receive immediate feedback on their performance, which is crucial for skill development, limited feedback can be a disadvantage. The quality of simulation scenarios and online courses can vary widely, affecting the effectiveness of learning. Evaluating learning outcomes in a technology-rich environment can be complex.

It is essential to carefully consider these potential disadvantages and implement strategies to mitigate their impact. A balanced approach that combines traditional methods with innovative technologies can optimize the educational experience for orthopaedic trainees. Planning a combined approach to traditional learning and including technological advantages can be the best approach in these modern times.

While innovative educational methods such as simulation, virtual reality, and online learning offer significant advantages in orthopaedic education and training, they also present certain challenges and potential disadvantages, such as high costs of infrastructure and content development. Implementing simulation labs and VR technology requires substantial financial investment in equipment, software, and maintenance. Creating high-quality simulation scenarios and online courses demands significant time, expertise, and resources. Technical difficulties with simulation equipment or online platforms can disrupt learning and training. Access to technology and internet connectivity may vary among trainees, creating disparities in learning opportunities. Simulation and virtual environments may not fully replicate the tactile sensations of real-life surgical procedures.

Excessive reliance on simulation could potentially reduce opportunities for hands-on surgical experience. Online learning and independent simulation practice can lead to reduced interaction with peers and mentors and can lead to isolation and reduced interaction. As trainees may not receive immediate feedback on their performance, which is crucial for skill development, limited feedback can be a disadvantage. The quality of simulation scenarios and online courses can vary widely, affecting the effectiveness of learning. Evaluating learning outcomes in a technology-rich environment can be complex.

It is essential to carefully consider these potential disadvantages and implement strategies to mitigate their impact. A balanced approach that combines traditional methods with innovative technologies can optimize the educational experience for orthopaedic trainees. Planning a combined approach to traditional learning and including technological advantages can be the best approach in these modern times.

While innovative educational methods such as simulation, virtual reality, and online learning offer significant advantages in orthopaedic education and training, they also present certain challenges and potential disadvantages, such as high costs of infrastructure and content development. Implementing simulation labs and VR technology requires substantial financial investment in equipment, software, and maintenance. Creating high-quality simulation scenarios and online courses demands significant time, expertise, and resources. Technical difficulties with simulation equipment or online platforms can disrupt learning and training. Access to technology and internet connectivity may vary among trainees, creating disparities in learning opportunities. Simulation and virtual environments may not fully replicate the tactile sensations of real-life surgical procedures.

Excessive reliance on simulation could potentially reduce opportunities for hands-on surgical experience. Online learning and independent simulation practice can lead to reduced interaction with peers and mentors and can lead to isolation and reduced interaction. As trainees may not receive immediate feedback on their performance, which is crucial for skill development, limited feedback can be a disadvantage. The quality of simulation scenarios and online courses can vary widely, affecting the effectiveness of learning. Evaluating learning outcomes in a technology-rich environment can be complex.

It is essential to carefully consider these potential disadvantages and implement strategies to mitigate their impact. A balanced approach that combines traditional methods with innovative technologies can optimize the educational experience for orthopaedic trainees. Planning a combined approach to traditional learning and including technological advantages can be the best approach in these modern times.

Nevertheless, this should not stop us from taking a step forward towards a better technique of learning. One can think of a common center for installing such advanced instruments and hands-on experiences where resident doctors or trainees can come together from different institutions and learn the skills, all at a minimal cost in a well-spread schedule programmed by the institutions. In this way, the hurdles of this learning can be managed, and all get an equal opportunity to develop the skills.

The curriculum needs to change as teaching strategies do. In order to teach future surgeons to incorporate developing technology like robotics and artificial intelligence into their practice, these elements should be included. The fundamentals of surgical technique, patient care, and moral decision-making must continue to be at the center of the curriculum.

Not all medical institutions are equally funded, have the same patient inflow, or have complex surgeries performed at their centers. Imbibing these newer technological advances as a mode of learning would help create a platform where all the trainees or residents can come and exchange their ideas and practice these skills, which might not be available back in their centers due to variable limitations.

It is also imperative to prioritize lifetime learning skills. In order to stay up-to-date with the latest advancements in the field and ensure that their patients receive the best care possible, orthopaedic surgeons will need to engage in ongoing education.

To conclude, orthopaedics has a bright future, but maintaining quality and equal standards across all institutions demands a dedication to innovation in training and education. Through the adoption of innovative technology, efficient use of skills in laboratories, and curriculum adaptation, we can equip upcoming generations of orthopaedic surgeons to provide the best possible care to a constantly evolving patient population. This approach would help maintain a certain standard in the training of residents, which would benefit all the patients in the end.
